# Molecular and Clinical Characterization of Invasive *Streptococcus pyogenes* Isolates: Insights from Two Northern-Italy Centers

**DOI:** 10.3390/pathogens14020152

**Published:** 2025-02-05

**Authors:** Carmelo Bonomo, Eva Mannino, Dafne Bongiorno, Caterina Vocale, Armando Amicucci, Dalida Bivona, Davide Guariglia, Emanuele Nicitra, Grete Francesca Privitera, Giuseppe Sangiorgio, Stefania Stefani, Simone Ambretti

**Affiliations:** 1 Department of Biomedical and Biotechnological Sciences (BIOMETEC), University of Catania, 95124 Catania, Italy; carmelo.bonomo@phd.unict.it (C.B.); dalida.bivona@phd.unict.it (D.B.); emanuele.nicitra@phd.unict.it (E.N.); giuseppe.sangiorgio@phd.unict.it (G.S.); stefania.stefani@unict.it (S.S.); 2 Department of Public Health, Experimental and Forensic Medicine, University of Pavia, 27100 Pavia, Italy; eva.mannino01@universitadipavia.it; 3 Microbiology Unit, IRCCS Azienda Ospedaliero-Universitaria di Bologna, 40138 Bologna, Italy; caterina.vocale@aosp.bo.it (C.V.); simone.ambretti3@unibo.it (S.A.); 4 Department of Medical and Surgical Sciences, Section of Microbiology, University of Bologna, 40136 Bologna, Italy; armando.amicucci85@gmail.com (A.A.); davide.guariglia@studio.unibo.it (D.G.); 5 Department of Clinical and Experimental Medicine, Department of Mathematical and Computational Science, University of Catania, 95124 Catania, Italy; greteprivitera@gmail.com

**Keywords:** *Streptococcus pyogenes*, invasive group A Streptococcus (iGAS), whole genome sequencing (WGS)

## Abstract

*Streptococcus pyogenes* (Group A Streptococcus, GAS) is a Gram-positive pathogen responsible for both superficial and invasive infections (iGAS), with increasing global incidence in recent years. This study aims to characterize the molecular and clinical features of iGAS cases in Bologna and Imola (Italy) between 2022 and 2024. Thirty-five invasive isolates were analyzed through whole-genome sequencing (WGS) to investigate the distribution of emm types, antimicrobial resistance (AMR) genes, and virulence factors. Clinical and epidemiological data were retrospectively collected and analyzed. The majority of cases (80%) were recorded in 2023, predominantly among patients aged over 65 (60%). Bloodstream infections were present in 97.1% of cases, and comorbidities such as diabetes and immunosuppression were common. Empirical antibiotic therapy often involved penicillin/β-lactam inhibitors, while oxazolidinones were the most frequently used in targeted regimens. The in-hospital mortality rate was 20%. Genomic analysis identified *emm*1, *emm*12, and *emm*89 as the most prevalent types, associated with specific virulence profiles and resistance determinants. This study highlights the critical role of *emm* typing and genomic characterization in understanding the pathogenicity of GAS. These findings contribute to the identification of risk factors for severe outcomes and underscore the need for targeted prevention and treatment strategies in vulnerable populations.

## 1. Introduction

*Streptococcus pyogenes* (Group A Streptococcus, GAS) is a Gram-positive pathogen responsible for a wide range of diseases, including superficial infections such as pharyngitis and impetigo, as well as severe invasive infections (iGAS) like necrotizing fasciitis, streptococcal toxic shock syndrome, and bacteremia [1,2]. The clinical outcomes of GAS infections can vary widely, with invasive infections being associated with significant morbidity and mortality, particularly in vulnerable populations such as children, the elderly, and immunocompromised individuals [3]. Epidemiologically, the diversity of circulating *emm* types plays a pivotal role in the clinical manifestations and spread of GAS infections. Common *emm* types, such as *emm*1, *emm*12, and *emm*28, are frequently associated with invasive infections, while others may be more prevalent in non-invasive conditions [4,5]. Notably, *emm*1 strains are often implicated in severe outcomes due to their enhanced production of key virulence factors, such as streptococcal pyrogenic exotoxins and streptolysins [6,7]. Superantigens, such as SpeC, SpeJ, and SmeZ, have been commonly observed in isolates belonging to *emm*12 mainly responsible for scarlet fever, impetigo, and pharyngitis [8]. *emm*28, instead, is frequently associated with invasive infections in young women and puerperal infections [9,10]. In recent years, a concerning increase in iGAS cases has been reported globally [11], with notable surges in regions such as Europe, North America, and Australia [12,13,14]. For example, the UK has observed periodic outbreaks of iGAS, with *emm*1, *emm*12, and *emm*89 being dominant during these episodes [15,16]. Similarly, recent reports from the United States and Canada indicate rising incidence rates among children, linked to a resurgence of certain *emm* types [17,18,19,20,21]. Moreover, several studies have reported similar trends in Italy [22,23,24,25,26] with a prevalence of pediatric cases. This increase has been partially attributed to post-pandemic shifts in population immunity, changes in healthcare access, and evolving strain characteristics [27]. This study focuses on the molecular characterization of iGAS isolates collected in Bologna and Imola (Italy) from 2022 to 2024. By examining the distribution of antimicrobial resistance genes, virulence factors, *emm* types and *emm* clusters, the research aims to uncover potential associations between specific genomic features and the severity of clinical outcomes. Moreover, the genomic correlation between the isolates has been investigated to detect possible clusters. Understanding these relationships is critical for elucidating the mechanisms underlying the invasiveness of GAS infections and guiding future strategies for prevention and management.

## 2. Materials and Methods

### 2.1. Sample Description and Clinical Data

The present study includes a total of 35 invasive *S. pyogenes* isolates (iGAS) collected between 2022 and 2024 from hospitals located in Bologna and Imola (Italy). Clinical and epidemiological data were retrospectively collected from hospital records and databases reported as frequencies and percentages or as medians and interquartile ranges (IQRs) to describe the baseline characteristics when appropriate. Descriptive analyses were performed using R version 3.6.0 [28].

### 2.2. Bacterial Growth Conditions

*Streptococcus pyogenes* isolates were cultured on blood agar and incubated overnight at 35–37 °C in an atmosphere enriched with 5% CO_2_ under anaerobic conditions.

### 2.3. Whole Genome Sequencing (WGS)

Genomic DNA was extracted and Next-Generation Sequencing (NGS) was performed on the Illumina MiSeq (Illumina Inc., San Diego, CA, USA) platform, following previously described methods [29]. Prior to genomic DNA extraction, identification was confirmed by mass spectrometry (MALDI-TOF) employing the Maldi Biotyper Sirius System (Bruker, Billerica, MA, USA).

### 2.4. Bioinformatic Analysis

Thirty-five paired-end bacterial raw reads were firstly trimmed with TrimGalore (v0.6.10) [30,31] to remove the adapter sequence and then assembled de novo using SPAdes (v.4.0.0) [32]. Draft genome quality was assessed using Fastqc (https://www.bioinformatics.babraham.ac.uk/projects/fastqc/, accessed on 16 April 2024). The fasta built through SPAdes have been inspected for the research of phage-associated virulence factors employing the software geNomad (v.1.8.0) [33]. AMR Genes and Chromosomal Virulence factors have been studied employing several tools: AMRfinder plus (v.3.12.8 with database version 2024-05-02.2) [34], Abricate (v.1.0.1) [35], with the databases NCBI [36], CARD [37], Resfinder [38], ARG-ANNOT [39], MEGARES [40], VFDB [41], and virulencefinder (v2.0) [42]. An 80% quality threshold was set for coverage and identity for AMR genes detection. The ST was calculated with mlst software (v.2.23.0) [43] which employs the PubMLST database [44]. *emm* cluster and *emm* type have been inspected with emmtyper software (v.0.2.0) [45,46]. A phylogenetic tree was constructed using mafft (v7.525) [47] with 100 iterations, followed by fasttree (v.2.1.11) [48]. The tree was plotted using the ggtree (v.3.12.0) [49,50] R package. The Heatmap has been created using the R packages ComplexHeatmap (v.2.20.0) [51].

## 3. Results

### 3.1. Clinical Results

Between 2022 and 2024, our study identified a total of 35 cases of Group A Streptococcus (GAS) infections. The distribution of these cases varied over the years, with the majority occurring in 2023. Specifically, 28 cases (80.0%) were recorded in 2023, while 3 cases (8.6%) were noted in 2022 and 4 cases (11.4%) in 2024. This temporal spread highlights a significant peak in GAS infections during 2023. The study population consisted of 35 patients with an almost equal gender distribution: 18 females (51.4%) and 17 males (48.6%). The age demographics revealed that the majority of patients were adults over the age of 65, accounting for 21 individuals (60.0%). This was followed by those aged between 35 and 65 years, comprising 9 patients (25.7%). Only a small fraction, specifically three patients (8.6%), were under the age of 18. Geographically, most cases were concentrated in Bologna, with 31 cases (88.6%), while the remaining 4 cases (11.4%) were from Imola. In terms of healthcare settings, the distribution suggests that GAS infections often present as emergencies requiring immediate attention; in fact, the Emergency department handled the largest number of cases, managing 22 patients (62.9%). The initial site of infection varied among patients. The skin was the most common starting point for infections, seen in 17 cases (48.6%). Other notable sites included the oral cavity with four cases (11.4%) and otitis with three cases (8.6%). Bloodstream infections (BSI) were prevalent in nearly all cases, affecting 34 patients (97.1%), underscoring the severity and systemic nature of these infections. Regarding patient comorbidities, more than half of the patients had significant health issues as indicated by a high Charlson Comorbidity Index score (≥5) in 19 patients (54.3%). Diabetes was present in twelve patients (34.3%), and seven patients (20.0%) were immunocompromised, adding complexity to their treatment and management. Moreover, severe outcomes requiring a secondary admission to ICU were reported in 10 cases (28.6%). Treatment regimens varied between empiric and targeted approaches. For empiric therapy, penicillin/β-lactam inhibitors were most commonly used, administered to 18 patients (51.4%), followed by mixed antibiotic regimens for 12 patients (34.3%). For targeted therapy, oxazolidinones were used most frequently in ten cases (28.6%), followed by penicillin/β-lactam inhibitors and cephalosporins, each used in eight cases (22.9%). An in-hospital mortality rate of 20.0% was also reported, reflecting the serious nature of GAS infections and the challenges associated with their treatment in vulnerable populations such as older adults with multiple comorbidities. A detailed description of the demographic of the study population is shown in Table 1. Supplementary Materials (Table S1) show detailed clinical features related to each isolate.

### 3.2. Bacterial Typing

Among the 35 isolates analyzed, 15 different *emm* types were identified. Multi-locus sequence typing (MLST) revealed a unique sequence type (ST) for each *emm* type, except for ST28, which was associated with both *emm* 1.0 and the subtype *emm* 1.3.

These two *emm* types represent the most common ones, in our sample. Specifically, *emm* type 1.0 was reported for 9 strains (25.6%), whereas *emm* 1.3 was identified in 4 strains (11.14%). These were followed by *emm* type 12 (ST36) and *emm* type 28 (ST458), each of them detected in three strains (8.6%). Other *emm* types included *emm* 75 (ST49), *emm* 87 (ST62), *emm* 89 (ST101), *emm* 90.2 (ST184) and *emm* 3.93 (ST315), each of them represented by two isolates (5.7%). Lastly, the following *emm* types were identified in a single isolate (2.8%) each: *emm* 22 (ST47), *emm* 76.0 (ST50), *emm* 8.0 (ST58), *emm* 63 (ST210), *emm* 77.0 (ST399), and *emm* 480.0 (ST506).

The temporal distribution of the different *emm* types detected was analyzed. In detail, *emm* 90.2 was found to be prevalent during 2022 with 67% of the cases, while *emm* 77.0 accounted for 33% of the distribution. In 2023 there was a more heterogeneous distribution of *emm* types. The most common is *emm* 1.0 with 32% of the cases, followed by subtype *emm* 1.3, with 14%. Other notable types are *emm* 12 (11%), *emm* 28 (11%), *emm* 89 (7%), *emm* 480.0 (4%) and several smaller *emm* types between 4% and 7%, including *emm* 22, *emm* 76.0, *emm* 75 and *emm* 63. In 2024, most cases (56%) were identified as *emm* 3.93, followed by *emm* 63 with 25% and *emm* 75 accounted for the remaining 25%. During the three years under study, only *emm* 75 was detected in two different years, 2023 and 2024.

### 3.3. Antimicrobial Resistance and Virulence Genes

A total of 33 virulence factors were identified. Of these, 27 are localized on the chromosome and the remaining 6 are phage-associated.

The surface proteins identified included fibronectin-binding proteins *fbp*54, present in 34/35 strains (97.1%), *cpa*, present in 15/35 strains (42.8%) and *fba*A, present only in one strain, and laminin-binding protein *lemb*, present in all strains (100%).

Among the capsule synthesis genes, *has*A is present in 31 out of 35 isolates (88.6%).

The Dnase *mf/spd* is detected in 34/35 strains (97.1%) and the peptidase *scp*A in 30/35 strains (85.7%). As for the extracellular toxins *slo*, coding for streptolysin O, is present in all strains. The streptococcal exotoxin precursors *spe*B and *spe*G are also present in 35/35 strains (100%) and 34/35 strains (97.1%), respectively.

Other streptococcal exotoxin precursors *spe*A and *spe*C are phage-associated virulence factors; the first was detected in 17/35 strains (48.6%) and the second in 15/35 strains (42.8%). Moreover, *spe*A is only present in some *emm* types (1.0, 1.3, 3.93, and 90.2).

In terms of antimicrobial resistance (AMR) genes, four genes (*lmp*R, *erm*B, *tet*M, and *lmp*RI) were detected. All strains (100%) carry the *lmr*P gene. Additionally, three strains carry *erm*B, *tet*M, and *lmp*RI, two strains carry *tet*M and *lmp*RI, and one strain carries only *tet*M.

**Figure 1:**
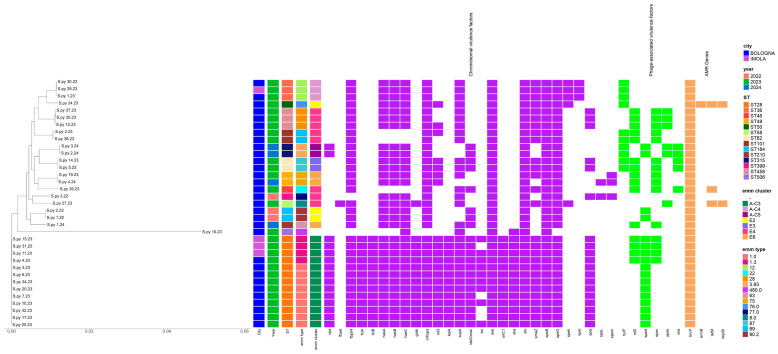
Phylogenetic relatedness and genomic features of iGAS isolates, including chromosomal (purple) and phage-associated (green) virulence factors and AMR genes (orange).

Figure 1 comprehensively summarizes all genomic features and a phylogenetic tree of the isolates included in the study.

## 4. Discussion

The present study examines the spread of iGAS infections in the Greater Bologna area (Italy) between 2022 and 2024, which was shown to be consistent with the increase observed in other geographic areas across the globe [52,53]. A total of 35 isolates were collected from different hospitals in Bologna and Imola and sent to the Molecular Microbiology and Antibiotic Resistance (MMAR) laboratory of the University of Catania to be characterized. Specifically, temporal trends, clinical characteristics, bacterial typing, and genomic features were determined by employing NGS technology. The temporal distribution of cases demonstrated a significant peak in 2023, accounting for 80% of the infections. This observation may reflect increased transmission, enhanced detection, or outbreak dynamics during that year. In contrast to other studies [54,55] which reported a prevalence of iGAS in children, older adults (60% > 65 years) are predominant in our study likely due to age-related immunesenescence and comorbidities. The high Charlson Comorbidity Index (≥5) in over half of the patients, coupled with a substantial prevalence of diabetes (34.3%) and immunocompromised states (20%), further illustrates the significant burden of GAS infections in high-risk populations, which is in line with prior studies [56,57]. The high prevalence of bloodstream infections (97.1%) and emergency department presentations (62.9%) highlights the severity of GAS infections, necessitating prompt recognition and aggressive management. Additionally, the predominance of skin infections (48.6%) as the primary infection site suggests the importance of this portal of entry in invasive disease. However, the high in-hospital mortality rate (20%) underscores the challenges of managing severe GAS infections, particularly in older adults with significant comorbidities.

The *emm* typing revealed a remarkable diversity, with 15 different *emm* types identified among the 35 isolates. This diversity reflects the capacity of GAS to adapt and circulate in diverse populations. The predominance of *emm* 1.0 (ST28) (25.6%) and its association with invasive disease aligns with previous studies linking *emm* 1.0 isolates to severe outcomes [58]. In contrast to other studies, suggesting an association of this serotype with the pediatric population, nearly 80% of its manifestations occurred in adults. Subtype *emm* 1.3 (ST28) (11.4%) and other types such as *emm* 12 (ST36) and *emm* 28 (ST458) were also frequently detected, with temporal heterogeneity noted in their distribution. Additionally, 2/3 of *emm* 12 isolates were associated with secondary BSI, despite other studies reported a negative correlation between this serotype and invasive infections [22,59].

Notably, only *emm* 75 (ST49) was detected across multiple years (2023 and 2024), suggesting its persistence or re-emergence. A similar pattern was shown in 2023 in Milan as reported by Mangioni et al. [22]. On the contrary, other documented outbreaks of iGAS infection in adults in recent years have been driven by specific *emm* types prevalent in hospital or long-term care facilities [60,61]. The temporal clustering of *emm* types (e.g., *emm* 90.2 in 2022 and *emm* 3.93 in 2024) may indicate localized outbreaks or shifts in strain circulation. This finding emphasizes the importance of continued surveillance to monitor trends and inform public health interventions.

The high prevalence of key virulence factors, including *slo* (streptolysin O), *speB*, and *speG*, underscores the pathogenic potential of these strains. The universal presence of *lemb* and the near-universal presence of *fbp54* and *mf/spd* highlight their critical roles in GAS adhesion and immune evasion. Interestingly, phage-associated exotoxins *speA* and *speC* were detected in 48.6% and 42.8% of strains, respectively, with *speA* restricted to specific *emm* types (1.0, 1.3, 3.93, and 90.2). This finding suggests potential links between prophage content and strain-specific virulence.

In terms of antimicrobial resistance (AMR), the universal presence of the *lmrP* gene (efflux pump) is notable, suggesting intrinsic resistance to specific antibiotics such as lincosamides, macrolides, streptogramins, and tetracyclines [62]. The totality of the isolates displayed susceptibility to penicillin confirming that β-lactams remain the first-line treatment option for both invasive and non-invasive GAS infections [63]. Interestingly, the detection of *ermB*, *tetM*, and *lmpRI* in a subset of strains highlights emerging resistance mechanisms, potentially limiting alternative treatment options (e.g., macrolides and lincosamides) in the presence of β-lactam-resistant strains. These findings reinforce the need for judicious antibiotic use and ongoing surveillance of AMR trends in GAS in order to avoid recurrent infections, treatment failure, and poor patient outcomes [2].

When comparing our findings to other Italian studies examining iGAS infections, several similarities and differences emerge. First, in terms of the population affected, both our study and the work by Mangioni et al. (2023) observed a higher incidence of iGAS in adults, especially in those with underlying comorbidities, contrasting with the pediatric predominance seen in earlier reports [46,47]. This shift may be attributed to factors like age-related immune decline and the resurgence of iGAS post-pandemic, similar to the pattern observed in other regions [52]. Additionally, the diverse distribution of *emm* types in our study, especially the prevalence of *emm* 1.0 and its significant association with invasive disease, aligns with global findings, yet differs in the proportion of infections occurring in adults rather than children, as traditionally expected for this serotype [58]. Moreover, while some studies have reported limited antimicrobial resistance in iGAS isolates [60], we noted emerging resistance in a subset of our strains, which is in line with findings from similar outbreaks [62]. This underscores the importance of vigilant surveillance and appropriate antimicrobial stewardship. Lastly, the involvement of phage-encoded exotoxins like SpeA and SpeC in our strains suggests a more complex relationship between strain-specific virulence and clinical outcomes, an aspect that warrants further exploration in future studies.

After the COVID-19 pandemic, invasive *Streptococcus pyogenes* infections (iGAS) re-emerged in Europe, reaching a higher level compared to the pre-pandemic period. This resurgence was preceded by a significant impact on *Streptococcus pyogenes* circulation and associated risks of invasive infections (iGAS), largely due to the near-null circulation of many common respiratory pathogens, including *Respiratory Syncytial Virus* (RSV), seasonal flu, and human herpesvirus 3 (HHV-3), during the pandemic [64]. The widespread use of face masks and increased attention to hygiene during the pandemic played a crucial role in this phenomenon. These respiratory viruses are well-known predisposing factors for *Streptococcus pyogenes* superinfections, which heighten the risks associated with invasive infections (iGAS) [23]. In addition, a lowered immunity to GAS may have contributed significantly to the post-pandemic increase in GAS infections, increasing, in turn, the probability of invasive manifestations [65].

In comparison to Massese et al., our study aligns with the observation of increased GAS infection after the pandemic; in contrast to our findings, they showed that the affected individuals were younger (preschoolers) than those affected previously (middle childhood individuals) [66]. A post-pandemic increase in Italy was also described by De Maio et al., although their study did not register a significantly increased incidence of invasive manifestations after COVID-19 [67].

The increased spread, especially after COVID-19, along with a high mortality rate and extensive bloodstream involvement, underscore the need for early recognition and aggressive management of GAS infections, particularly in older adults with comorbidities [68]. Preventive measures oriented towards the reduction in GAS dissemination within the general population are strictly required. In this context, genomic analyses are crucial for the identification of emerging strains showing remarkable virulence. Moreover, empiric therapy with penicillin/β-lactam inhibitors (51.4%) remains the established practice, although the use of targeted therapies, including oxazolidinones (28.6%), highlights the evolving role of precision medicine in managing GAS infections. The association between phage-encoded exotoxins and specific *emm* types deserves further investigation to understand their role in clinical outcomes.

## 5. Conclusions

The small sample size limits the statistical power of our findings; however, this study highlights the complexity of GAS infections, characterized by significant temporal variation, high clinical burden, and extensive genomic diversity. The findings underscore the importance of integrated clinical, epidemiological, and genomic surveillance and the need for a national surveillance program for GAS infections to guide effective prevention and management strategies. The lack of pediatric cases precludes any investigation regarding invasive disease in children, a demographic in which certain *emm* types may exhibit distinctive pathogenic behaviors. Future research should focus on elucidating the molecular basis of GAS pathogenesis, exploring novel therapeutic targets, and advancing vaccine development to reduce the global impact of GAS infections.

## Figures and Tables

**Table 1:** Demographic and clinical characteristics of the study population.

	N = 35	%
Year		
2022	3	8.6%
2023	28	80.0%
2024	4	11.4%
City		
Bologna	31	88.6%
Imola	4	11.4%
Ward		
Cardiology	3	8.6%
Emergency	22	62.9%
Geriatrics	1	2.9%
Hematology	1	2.9%
Infectious Disease	1	2.9%
Intensive Care	1	2.9%
Neonatology	1	2.9%
Neurosurgery	1	2.9%
Otorhinolaryngology	1	2.9%
Pediatrics	1	2.9%
Plastic Surgery Anesthesiology	1 1	2.9% 2.9%
Gender		
Female	18	51.4%
Male	17	48.6%
Bloodstream infection (BSI)		
No	1	2.9%
Yes	34	97.1%
Age		
<18 y	3	8.6%
18–35 y	2	5.7%
35–65 y	9	25.7%
>65 y	21	60.0%
Race		
White	32	91.4%
Black	1	2.9%
Mixed	2	5.7%
Diabetes		
No	23	65.7%
Yes	12	34.3%
Immunocompromised		
No	28	80.0%
Yes	7	20.0%
Urinary tract infection (UTI)		
No	32	91.4%
Yes	3	8.6%
Charlson Comorbidity Index (age-adjusted)		
Low (0–2)	8	22.9%
Moderate (2–4)	8	22.9%
High (≥5)	19	54.3%
Infection starting site		
Artritis	1	2.9%
Oral cavity	4	11.4%
Osteomyelitis	1	2.9%
Otitis	3	8.6%
Pharyngitis	2	5.7%
Prosthesis	1	2.9%
Skin Endocarditis	17 1	48.6% 2.9%
Unknown	5	14.3%
Secondary admission to Intensive care unit (ICU)		
No	25	71.4%
Yes	10	28.6%
In-hospital mortality		
No	28	80.0%
Yes	7	20.0%
Co-infection		
No	30	85.7%
Yes	5	14.3%
Empiric antibiotic therapy		
None	1	2.9%
Penicillin/B-latt in.	18	51.4%
Cephalosporin	3	8.6%
Carbapenems	1	2.9%
Mixed	12	34.3%
Targeted antibiotic therapy		
None	7	20.0%
Penicillin/B-latt in.	8	22.9%
Cephalosporin	8	22.9%
Quinolones	1	2.9%
Oxazolidones	10	28.6%
Mixed	1	2.9%

## Data Availability

The original contributions presented in the study are publicly available. These data can be found at: http://www.ncbi.nlm.nih.gov/bioproject/1193569 (accessed on 16 April 2024).

## Supplementary Materials

The following supporting information can be downloaded at: https://www.mdpi.com/article/10.3390/pathogens14020152/s1, Table S1: detailed clinical features of iGAS isolates.
